# The Pathophysiology and Management of Diabetic Ketoacidosis in COVID-19 Patients: a Literature Review

**DOI:** 10.2478/jccm-2021-0024

**Published:** 2021-11-13

**Authors:** Mariana Cornelia Tilinca, Maximilian Cosma Gliga, Andreea Varga

**Affiliations:** 1 George Emil Palade University of Medicine, Pharmacy, Science, and Technology of Targu Mures, Targu Mures, Romania; 2 Mures County Hospital, Targu Mures, Romania

**Keywords:** COVID-19, diabetes, ketoacidosis, pandemic, pathogenesis

## Abstract

Diabetic individuals are considered a vulnerable population during the COVID-19 Pandemic, and several studies noted worse outcomes, including death, among those who get infected. Diabetic emergencies, such as ketoacidosis (DKA), are common and potentially life-threatening conditions in uncontrolled patients. While the pathophysiological background of the relationship between COVID-19 and DKA is not fully understood, early reports available so far indicate that patients with pre-existing diabetes who get infected with the SARS-CoV 2 virus are at higher risk of DKA. It was also suggested that DKA is a poor prognostic sign for infected patients, these being at higher risk of developing worse forms of COVID-19 disease and having high mortality. Therefore, healthcare personnel dealing with such patients face a considerable challenge, as the correct and safe emergency management of such cases is far from established. This article aimed to conduct a study that reviews the current published data available about patients with DKA and COVID-19.

## Introduction

Diabetic ketoacidosis (DKA) is a well-known medical emergency occurring in uncontrolled diabetic patients, responsible for significant mortality and morbidity, therefore highlighting the vital importance of early diagnosis and treatment in managing these patients. Although more frequently seen in patients with Type 1 diabetes, considering the metabolic disorder background of absolute insulin deficiency, diabetic ketoacidosis is not uncommon in Type 2 diabetes patients. Prompt action is required whenever clinical suspicion is present [1,2]. As the prevalence of diabetes continues to grow, the latest data from the International Diabetes Federation estimated 463 million cases worldwide, compared to 285 million in 2010. It is expected that the morbidity and mortality of this condition are also increasing [3,4].

The novel-coronavirus that causes severe acute respiratory syndrome 2 (SARS-2) disease (COVID-19) emerged from Wuhan, China, in December 2019, leading to a global pandemic, with more than 44 million cases and over 1.1 million deaths confirmed as of 28.10.2020 [5,6]. Despite many studies indicating that diabetes is a significant risk factor contributing to the severity and mortality of COVID-19 disease, the pathophysiological relationship between them remains unclear. Recent observations suggested that there is a bidirectional relationship between diabetes and COVID-19, as diabetic patients are known to be at an increased risk of developing severe forms of COVID-19, while on the other hand, acute complications like DKA or hyperosmolar hyperglycaemic state (HHS) frequently develop with an underlying acute infection like COVID-19 [7,8,9]. Other indirect adverse effects of the Pandemic on diabetic patients are the increasing burden that health care systems face as the epidemiological situations in various countries get out of control, leading to large numbers of non-COVID-19 patients left without the medical care they would require. Consequently, many diabetic patients who missed their timely medical visits are at increased risk of developing metabolic imbalances leading to many of them presenting to emergency departments with acute life-threatening complications like DKA.

Furthermore, lack of physical activity because of lockdowns imposed by the government in many countries might be another negative factor for diabetic patients, as sedentarism plays a significant role in the progression of disease severity and metabolic imbalance [7,10].

Considering all these aspects, both the healthcare personnel dealing with diabetic patients and those on the front-line of the pandemic caring for COVID-19 patients should be aware of the challenges in managing diabetic patients during these times, paying careful attention to acute complications such as DKA or HHS. Also, new patients presenting with DKA should be carefully screened for COVID-19, which could be a possible infectious triggering factor of the metabolic imbalance [11,12].

There little data available regarding the pathophysiological relationship between diabetic ketoacidosis and COVID-19 and the impact of the pandemic on the evolution of acute diabetic complications.

The study aimed to review current evidence about the epidemiology, pathophysiological mechanisms, and diagnostic and management challenges of DKA during the COVID-19 pandemic.

## Method

A literature search was performed using “COVID-19”, “diabetes”, and “diabetic ketoacidosis” as keywords through the “PubMed” and “Google-Scholar” search engines.

## Diabetes and COVID-19

The first published studies about the evolution and prognosis of diabetic patients with COVID-19 originate from China. It was reported that COVID-19 patients who had diabetes had higher hospital admission rates and developed worse forms of pneumonia than infected patients without previous medical conditions. Another study reported that diabetes was the second most frequent comorbidity after hypertension in a sample of 140 patients diagnosed with Covid-19 and hospitalised in Wuhan [13]. These observations have motivated researchers to study the pathophysiological changes responsible for the worse prognosis of COVID-19 patients with underlying diabetes. One suggested mechanism involves the compromised immune system of patients with uncontrolled glycaemic status, innate and adaptive humoral immunity, leading to a faulty first-line defence against infectious agents. Another possible explanation of this unfortunate relationship between diabetes and COVID-19 lies in the pro-inflammatory state, common in uncontrolled diabetic patients, which causes abnormal production of interleukin-6 (IL-6), and exaggerated cytokine responses frequently met in critically ill COVID-19 patients. Based on this previous hypothesis, it has been suggested that diabetic patients are at higher risk to develop a cytokine storm with all the multi-organ damaging consequences when suffering from COVID-19 [14,15].

## Current evidence on DKA during the COVID-19 pandemic

Besides the previously discussed, more commonly studied pathophysiological relationships between COVID-19 and diabetes, another important cause of concern when dealing with diabetic patients during the COVID-19 pandemic lies in managing acute hyperglycaemic complications DKA. DKA is the most common acute metabolic complication of diabetes caused by a relative or absolute insulin deficiency, which leads to reduced glucose uptake and utilisation by cells and excessive lipolysis. This further causes uncontrolled production of ketone bodies and a state of acidosis. Although DKA is an inflammatory state, it is commonly triggered and associated with an underlying acute illness, such as an infectious disease [16]. Recently, hospitalisation for DKA has seen an increasing trend; reports from a US study showing a 30% increase in hospital stay days during the last decade, while in less developed countries, the numbers are even higher, with mortality rates over 30%. Although it was previously considered a specific complication of Type-1 diabetes, some current evidence revealed that about 30% of hospital admissions for DKA involves patients with Type-2 diabetes [17].

More retrospective studies, case reports and a series of cases have observed that DKA is not an uncommon occurrence among hospitalised COVID-19 patients, which could be one of the causes of the higher mortality among these patients. In a Chinese study, almost half of the Covid-19 hospitalised patients died; all had medium and severe forms of Covid 19, with diabetes as a comorbidity and a 39% mortality in the studied cohort [18].

The first reports of DKA in COVID-19 patients outside China were by Croft A et al. (2020), who published the evolution of five new-onset DKA in a series of patients with Type 2 diabetes hospitalised for COVID-19. The authors highlighted the unusual severity of DKA in these cases of previously well-controlled diabetic patients, suggesting the significant negative impact of the novel coronavirus infection on insulin regulation and metabolic balance [19]. A recently published systematic review on DKA in COVID-19 patients studied the outcomes of a total of 110 SARS-CoV 2 infected patients who developed DKA during the hospital stay or presented with the typical features on admission [20]. Among these, 83% had DKA alone, while 17% had DKA and HHS. Seventy-seven per cent of the patients had pre-existing Type-2 diabetes. The total mortality rate was 45%, being higher for the group with combined DKA and HHS (69%) than those with DKA alone (29%). The results of this review also noted that the majority of patients were male (67%), and although the role of gender as a risk factor for DKA is controversial [21,22], some studies found males to be associated with worse outcomes in COVID-19 disease [23]. A significant predictor of in-hospital mortality found in this study was low pH on admission, while glucose levels were not associated with increased mortality or other clinical outcomes.

In cross-sectional UK study on 218 patients admitted with laboratory-confirmed COVID-19, pre-existing Type-2 diabetes was found in 61 patients (28%) and Type-1 diabetes in 6 (2.8%) patients. In this cohort, four patients (1.8%) presented DKA during the hospital stay, three of them having pre-existing Type-2 diabetes, and one was a newly diagnosed case based on high glycated haemoglobin C (HbA1C) and glucose levels [24]. A study of a Chinese cohort of 658 COVID-19 hospitalised patients reported that 42 (6.4%) patients had ketosis based on elevated urine or serum ketones, while seven patients (3%) had confirmed DKA based on the American Diabetes Association (ADA) criteria. Half of these patients had been previously diagnosed as having diabetes, and of the patients with DKA, two patients had underlying Type 2 diabetes while one had Type 1 diabetes [25]. An interesting finding of this study is that the patients with ketosis, compared to the rest of the cohort, had higher rates of acute respiratory distress syndrome (ARDS), and a significantly higher number of them eventually required mechanical ventilation. Mortality was also higher in the group with ketosis (21.4%) compared to the patients without (8.9%). These findings suggest a possible pathophysiological relationship between the metabolic state caused by the elevated ketone bodies in serum and worse COVID-19 disease evolution.

Besides DKA, another life-threatening acute metabolic complication of diabetes is HHS. Although the two conditions can be seen distinctively, both can sometimes overlap, especially in elderly, uncontrolled Type 2 diabetes patients [26,27]. As this category of patients is known to be the most vulnerable to the COVID-19 infection, considering the many associated comorbidities these patients usually have, recently, attention has been paid to combined DKA and HHS clinical outcomes in COVID-19 patients.

A retrospective study reported six cases with both DK and HHS and positive SARS-CoV 2 tests admitted to an intensive care unit in New York, USA [28]. All six cases were males, and a notable finding was that all patients presented mainly with DKA and HHS symptoms rather than specific COVID-19 respiratory manifestations. The SARS-CoV 2 infection was detected because the hospital was situated in an epicentre of the pandemic where all patients admitted were screened for the COVID-19 infection. Only one of the six patients had no previous diagnosis of diabetes, while the other five had underlying Type-2 diabetes. Out of the six patients, four patients died from respiratory failure or cardiac arrest, three of them while on mechanical ventilation. The mortality for this series of patients was high (67%), suggesting that the combination of HHS and DKA could have higher mortality in COVID-19 patients than isolated DKA or HHS.

## Molecular mechanisms involved in the pathogenesis of DKA and COVID-19 infection

The exact underlying mechanisms of how SARS-CoV 2 infection could accelerate DKA development, or how DKA contributes to a more severe evolution of the COVID-19 disease, leading to worse clinical outcomes and higher mortality, are still a subject of debate. The novel SARS-CoV 2 coronavirus belongs to a family of coronaviruses that have previously caused two significant outbreaks, one in the 2002-2004 SARS-1 outbreak and another, but with a higher mortality rate of MERS in 2012 [29,30]. Several studies observed a worse evolution in infected diabetic patients during these two previous coronavirus epidemics. The dysfunctional immune response in diabetic patients with increased production of Interleukin-17 that predisposes to a more severe lung injury was assumed to be the pathophysiological background resulting in the worse prognosis of these patients [31,32]. The current evidence on the novel coronavirus SARS-CoV 2 established that the S-protein binds to the Angiotensin-Converting Enzyme 2 (ACE-2) receptor, similar to SARS-CoV 1. These ACE-2 receptors are expressed in various degrees in the lungs, leading to the specific pathological changes and subsequent lung injuries of this infection. However, besides the lungs, ACE-2 receptors are also known to be expressed in other organs, including the pancreas. One of the main known functions of ACE-2 is to degrade Angiotensin-2 into Angiotensin 1-7. The physiological role of Angiotensin 2 is to produce inhibition of insulin secretion and reduce the vascularisation of the pancreatic islet cells, resulting in a hyperglycaemic state and a local pancreatic inflammation.

Angiotensin 1-7 has an antagonist role compared to Angiotensin 2 by stimulating insulin production and vasodilatation [33,34,35]. As the novel coronavirus has the ACE-2 as the target receptor, a downregulation of ACE-2 could occur in infected individuals, leading to a lack of counter-regulation to Angiotensin 2 effects, thus potentially producing a metabolic imbalance favouring a hyperglycaemic state [36]

This mechanism might explain the possible risk of COVID-19 patients developing DKA and metabolic deterioration. Further studies are needed to understand the complex role of the COVID-19 infection on pancreatic endocrine function and its consequences. Increased insulin resistance during COVID-19 infection might also be triggered by the frequently used medication to treat these patients, especially corticosteroids, but also remdesivir, lopinavir/ritonavir darunavir/cobicistat and interferon-b1. Cortisol, a potent endogenous stress hormone, is a known glucose elevating agent, states of hypercortisolism being frequently a cause for the metabolic deterioration of diabetic patients. Therefore, it could be hypothesised that cortisol, together with other stress hormones like catecholamines, might be involved in the vicious cycle of diabetic patients with COVID-19 during the hyper-inflammatory stress response phase [20, 36]. Another important characteristic of COVID-19 infection and DKA independent of the accompanying infection is the pro-inflammatory state with elevated levels of inflammatory markers. The elevation of the IL-6 marker is correlated with a maladaptive immune response in COVID-19 patients, leading to worse outcomes and higher mortality. Some studies also suggested IL-6 would be associated with ketosis independent of the COVID-19 infection, as it was found to be elevated in patients with DKA [37,38].

Further studies are needed to evaluate the role of other possible markers of disease severity, like pro-inflammatory cytokines or Vascular Endothelial Growth Factor (VEGF), which is known to be a predictor of poor outcome in ARDS [39]. Some studies noticed that obesity would pose an additional risk of severe complications and increased mortality for COVID-19 patients [40]. Several mechanisms could be behind this relationship between obesity and severity of COVID-19 disease, like the impaired respiratory function due to increased abdominal pressure, complications of the metabolic syndrome or secondary to the increased pro-inflammatory cytokines released from the adipose tissue [41]. Cardiovascular diseases are frequently associated with Type 2 diabetes, especially in uncontrolled patients who are more likely to develop DKA [42,43]. Several studies noted that cardiovascular complications, like myocardial injury, myocarditis, heart failure, are not uncommon among COVID-19 patients. Moreover, patients with pre-existing cardiac comorbidities, like hypertension, are at increased risk of suffering from these complications and having worse outcomes [44,45]. Further studies are needed to clarify the possibility of a synergistic relationship between the inflammatory cascades of COVID-19 and DKA and whether these are responsible for a worse prognosis in these patients.

## Update on the management of DKA during the COVID-19 pandemic

As recommended by the latest guidelines, the key components of DKA management include fluid and potassium replacement, intravenous insulin therapy and close monitoring of serum ketones, electrolytes, as well as acid-base status. It is recommended that patients continue long-acting analogue insulin therapy alongside intravenous infusions to prevent rebound hyperglycaemia after the intravenous treatment has ceased [1,46].

Management of DKA during the COVID-19 pandemic poses a significant challenge, as there is no current consensus or guidelines for this issue. In the case of compliant and clinically stable patients, where home testing of ketones is available, early treatment and prevention of this condition could be, in theory, done at home with self-hydration with oral fluids and following the therapy instructions by the healthcare team [47]. This could be an appealing option during the pandemic to prevent further exposure and risk of contamination. However, when there is a clinical suspicion of a worsening condition or a clinical or epidemiological picture suggestive of COVID-19 infection, urgent evaluation in a hospital setting is mandatory [38].

A known helpful strategy for managing uncomplicated and mild DKA that could also be used during the COVID-19 pandemic consists of the utilisation of subcutaneous insulin therapy; this would reduce the need for prolonged direct contact with healthcare personnel and nursing time. A. Cochrane review evaluated five randomised clinical trials concluded that subcutaneous rapid-acting insulin therapy is not inferior to intravenous insulin therapy for the treatment of mild DKA [48]. However, the role of subcutaneous insulin therapy for DKA should be reserved for mild cases with no complicated underlying diseases like acute kidney injury, myocardial infarction or stroke. Such cases would require an intensive-care unit setting and the use of intravenous therapy with constant biochemical and hemodynamic monitoring [1,49]. In case of severe forms of DKA, the optimal management should be done in an intensive care unit with intravenous insulin therapy and frequent monitoring of glucose, potassium and fluid balance. Currently, there is no consensus regarding insulin requirements in COVID-19 patients with DKA. From the little clinical evidence available, it would seem that insulin requirements in DKA patients may require up to 4 units/kg/ day, a dose that is higher than generally given to non-COVID patients.

The pro-inflammatory state of COVID-19 could explain this and the disturbance of the Angiotensin 1-7 counter-regulation of Angiotensin 2, leading to a higher degree of insulin resistance and possibly the accompanying organ damage involving the pancreatic islet cells. Careful attention should be paid to the concomitant use of corticosteroids and vasopressors in critically ill patients, as these could significantly influence the need for insulin. Therefore, a rapid adjustment method of insulin delivery to address fluctuations in glucose levels is vital for critically ill COVID-19 patients with DKA [50,51]. Another important issue of COVID-19 patients presenting with DKA is the restoration of potassium levels. By binding to the ACE-2 receptors, the SARS-CoV-2 infection could potentially lead to a state of hyperaldosteronism through reduced degradation of aldosterone, causing more severe hypokalaemia. Frequent and careful monitoring of serum potassium and quick interventions to restore it to normal levels are mandatory in COVID-19 patients with DKA, as hypokalaemia can worsen respiratory function through neuromuscular weakness [52,53]. DKA is also known to produce a hypercoagulable state, increasing the risk of thromboembolic events in these patients. This risk could be further enhanced by an underlying COVID-19 infection which is known to be associated with thromboembolic complications in severe cases. Considering this, it is vital to administer prophylaxis with low molecular weight heparin to all COVID-19 patients with DKA and keep a watchful eye on any signs or symptoms suggestive of a throm-boembolic event [54,55].

The nutrition of COVID-19 patients in ICU units is another matter of debate. There are no specific guidelines or data regarding the optimal nutritional support of COVID-19 diabetic patients. General recommendations for critical COVID-19 patients indicate that gastric enteral nutrition should be performed as early as possible after admission and should be preferred over parenteral nutrition. If gastric enteral nutrition is not possible or sufficient, parenteral nutrition (PN) can be prescribed using case-by-case decision-making. Risks of overfeeding and hyperglycaemia are to be taken into consideration, especially in diabetic patients. Liver function should also be closely monitored in all patients receiving gastric enteral nutrition or parenteral nutrition [56].

Another significant challenge in managing these patients lies in the administration of intravenous fluids. Although restoring fluid balance is a crucial aspect of treating ketosis, the lung involvement of COVID-19 infection could be worsened by increased extravascular lung water that would lead to severe hypoxia and potentially dangerous outcomes [57]. Therefore, excessive fluid administration should be avoided in patients with impaired respiratory function to prevent quick clinical deterioration. The latest UK guidelines by the National Inpatient Diabetes COVID-19 Response Group recommend a conservative approach to isotonic saline use. These suggest the administration of 250 ml fluid bolus for 15 minutes, with the further regulation of the infusion rate adapted to the patient’s weight

and pH. Patients with severe acidosis (pH < 7.1) should be treated more aggressively regarding fluid replacement than those with milder acidosis. The conservative approach to fluid replacement, although protecting the lungs, could lead to impaired clearance of ketones causing prolonged and worse ketonemia [58,59].

As DKA leads to a state of acidosis, patients frequently resort to respiratory-compensation tachypnoea; this state may be impaired in COVID-19 infected individuals.

Probably the most troublesome complication in all COVID-19 patients is acute hypoxemic respiratory failure [60]. The management of this has been a subject of debate, the choice of the therapeutical options depending on the severity of the lung disease and the general metabolic and hemodynamic status of each patient. The most conservative approach would be classic oxygen therapy, with the most recent guidelines recommending a target oxygen saturation between 9296%. However, a significant number of patients might require further respiratory support beyond the oxygen supplementation, the options ranging from high-flow nasal cannula non-invasive positive pressure ventilation or early intubation. The decision of whether to intubate patients with progressive respiratory failure early is still controversial, although some centres are switching from non-invasive positive pressure ventilation to techniques such as a high-flow nasal cannula or even early intubation.

The use of a high-flow nasal cannula is a viable option for avoiding or delaying intubation, providing, as it does, adequate pre-oxygenation before the need for intubation and mechanical ventilation [61].

The principles of respiratory support in COVID-19 respiratory failure are similar to those for ARDS caused by other diseases: low tidal volumes and driving pressures and adjusting the positive end-expiratory pressure depending on each patient’s requirements to protect the lungs. The addition of a severe metabolic complication like DKA makes the management and decision-strategy in the intensive-care unit handling of these patients even more problematic, as there is a lack of consensus and data regarding these issues [60, 62].

## Conclusions

Diabetic ketoacidosis management during the COVID-19 pandemic poses a significant challenge. Patients with pre-existing diabetes are considered a vulnerable group to SARS-CoV-2 related complications, and the virus could potentially lead to a worsening of glycaemic control and acute complications like DKA. Mortality in COVID-19 infected diabetic patients is high, resulting in DKA producing even worse clinical outcomes and prognosis. Close and continuous monitoring is mandatory for DKA patients with Covid-19, making it challenging for healthcare facilities to ensure a safe environment for both personnel and patients through enforcing strict protective and contact-limiting measures. Management of DKA patients with COVID-19 could be handled by aggressive intravenous insulin therapy and fluid restoration in some critically ill patients while taking a conservative approach to fluid administration to avoid excessive extravascular lung volumes that could worsen the respiratory function. Further research is needed to understand the pathogenesis of DKA during COVID-19 infection and to provide reliable guidelines on how to manage these patients.
